# Help for Our Sailors: The Mansion House Meeting

**Published:** 1917-07-14

**Authors:** 


### Help for our Sailors: The Mansion House Meeting.

UNDER the most favourable auspices the new Fund for the benefit of sailors was inaugurated at a well-attended and enthusiastic meeting at the Mansion House on the 5th inst. The Lord Mayor was supported by a large gathering of people who are well known in the shipping world and in connection with marine charities. The chief speakers were Sir John Jellicoe and Sir Edward Carson, and they paid a high tribute to the bravery and tenacity of British sailors, whether they belonged to the Royal Navy, the Royal Naval Reserve, or the mercantile marine.

The object of the Fund, incorporated by Royal Charter, is to secure more efficient aid for marine charities, to co-ordinate their work, to prevent overlapping, and generally to bring such influence and aid to bear that funds publicly supplied will be used to the best advantage for the good of sailors. The Fund is to be administered very much upon the same lines as King Edward's Hospital Fund for London; the institutions applying for participation will be inspected by independent visitors, the accounts and statistics are required to be kept on a uniform basis according to groups, showing in every instance the proportion of cost of management to that of maintenance, and a statistical record of the work done by every institution benefited will be published annually.

By command of the King, the Fund bears his name, the first president is Prince Albert, and the Duke of Connaught is the first chairman of the Council, which consists chiefly of prominent men in the shipping world. The hon. secretaries are the Marquis of Graham, Lord Inchcape, Sir Richard Williams-Bulkeley, and Mr. Charles Milburn. Capt. A. W. Clarke, an Elder Brother of Trinity House, who is the Deputy Chairman, announced at the meeting contributions amounting to £30,000, which list included twenty subscriptions from shipowners and shipping firms of £1,000 each. Prince Albert, in accepting the presidency, had sent a cheque for £100.

### SPEECH BY THE FIRST LORD OF THE ADMIRALTY.

(FROM THE "DAILY TELEGRAPH.")

Sir Edward Carson moved:

"That the loyal and humble duty of this meeting be conveyed to His Majesty the King, with their grateful appreciation of his command that the Fund be called 'King George's Fund for Sailors.'"

The other day, Sir Edward said, the Prime Minister stated that there was no harder-worked man in the land than the King. His concern for the welfare of his subjects, of course, goes without saying, and that he has devoted himself wholeheartedly to the care of those who are making such sacrifices for the country in this war is a matter which, I hope, the public in general thoroughly realise. We have revolutions in other countries. I believe in our country the war, which has brought all the units of our vast Dominions together, has added to the ever-increasing affection and loyalty towards His Majesty the King. (Cheers.) And the reason is not difficult to seek, because never has there reigned on any throne a more constitutional monarch. He has just paid a visit to the Fleet. The reception which met him there you have read of. But I am able, as First Lord of the Admiralty, to say that he himself could hardly appreciate the feeling of enthusiasm which the interest he showed in the Fleet, upon that occasion, has roused throughout the whole Service. He used to be called "Our Sailor Prince," as he truly was, and now he is "Our Sailor King." And he sends us to-day, in commanding that this Fund shall be called by his name, one more message of his interest in his sailor population, and his earnest prayer and desire that the nation may liberally and splendidly respond to the appeal which is now being made.

I am particularly glad that this Fund is to co-ordinate funds for all branches of sea service; that it is not for the Navy alone, but for the Naval Reserve, the Naval Volunteer Reserve, trawlers, mine-sweepers, auxiliaries of every kind, and the mercantile marine proper. It is sometimes said that the Admiralty do not show sufficient consideration for the mercantile marine, that we are a somewhat too autocratic people, who think a great deal of ourselves, and very little of others engaged on the sea. Any such conception of the Admiralty is absolutely false. For my own part, if there is one thing more than another which I am pleased at, in the evolution of this war, it is the way in which the mercantile marine, in all its branches, has been drawn closer and closer to the Royal Navy. I believe that this closeness is bound to grow. I believe that the proper evolution will be eventually that, more or less, it shall be all one Service. If we had not had the mercantile marine to fall back upon in this war, I really do not know where the country would be at the present moment. For my own part, whenever the time comes, when the question is ripe, whenever the mercantile marine (by which I mean not only the owners of merchant ships and captains, but the men in the Merchant Service) see fit to come into closer connection with the Navy, I do not think the difficulty of making that closer connection will arise at the Admiralty.

#### CHAPTERS OF HEROISM.

I spend a good deal of my time at the Admiralty reading from day to day the sacrifices of our naval auxiliaries and our mercantile marine. When the proper time comes for it to be published, you will have chapter after chapter of heroic deeds, of men of all ranks and all classes, in all branches of the Navy, the Navy auxiliary, and the mercantile marine. Take the mine-sweepers. Night and day they are carrying their lives in their hand to keep clear the pathways of the sea for the ships of commerce and the ships of war. I asked one man who had been torpedoed three times what he felt, what he did the third time. "I came ashore," was his reply, "and asked for another ship." (Cheers.) That is the grit that is going to see us through this war. (Cheers.) I want to bring it home to the public that, so far as I know, no man at the Admiralty grudges, in the slightest degree, not only the highest meed of honour which these men deserve, all desire that, in every way, the lot of the mine-sweeper and his dependants should be made equal to, ay, even better than, that of those who have made war the chief profession of their lives. Where can you get a charity, I do not call it a charity, a duty—(cheers)—which will come more readily home to your hearts than the duty of providing for sailors and their dependants?

Where can you get any occupation so necessary to the maintenance of the country and its people, so productive of wealth to the country, which so closely binds together all the different units that make up the British Empire? Make no mistake about it, but for the Navy and the Merchant Service you could have no British Empire such as it is to-day. Are we asking much that those who live through their efforts (and this includes every man, woman, and child in the kingdom) should give unsparingly in providing for these men and their dependants in the future. It is an elementary obligation, put upon every one of us, and if you were to look at it in the most selfish light you would ask yourselves, "What should I have in my pocket or my possession to-day if it were not for the Navy and the mercantile marine." (Hear, hear.) With all my heart I commend this new Fund. And I desire to express our respectful gratitude to His Majesty for having allowed the Fund to be called by his name. (Cheers.)

QUEEN ALEXANDRA'S LETTER.

Commodore Sir Richard Williams-Bulkeley moved a resolution thanking Queen Alexandra for her patronage of "Navy Week," commencing on July 23, during which a series of entertainments will be given by members of the theatrical profession in aid of the Fund. The speaker read a letter from Queen Alexandra, as follows:

"Please accept my thanks for your letter of the 27th ult., informing me of the proposal to hold a 'Navy Week,' for the benefit of our sailors and soldiers, to whom we owe so much. I need hardly tell you this movement has my most cordial sympathy and support, and I very gladly accede to your request for patronage. It is a source of much interest to me to hear that the members of the theatrical profession, who have done so much in the great cause of charity during this great war, are combining in this effort for our naval charities. I wish them all possible success in their undertaking." (Cheers.)

